# Death at no cost? Persons with no health insurance claims in the last year of life in Switzerland

**DOI:** 10.1186/s12913-018-2984-2

**Published:** 2018-03-14

**Authors:** Radoslaw Panczak, Viktor von Wyl, Oliver Reich, Xhyljeta Luta, Maud Maessen, Andreas E. Stuck, Claudia Berlin, Kurt Schmidlin, David C. Goodman, Matthias Egger, Kerri Clough-Gorr, Marcel Zwahlen

**Affiliations:** 1 Institute of Social and Preventive Medicine, University of Bern, Finkenhubelweg 11, 3012 Bern, Switzerland; 2 Epidemiology, Biostatistics & Prevention Institute, University of Zürich, Hirschengraben 84, 8001 Zurich, Switzerland; 3 Department of Health Sciences, Helsana Insurance Group, Palmstrasse 26b, 8401 Winterthur, Switzerland; 4 SWICA Gesunheitsorganisation, sante24, Winterthur, Switzerland; 5 University Center for Palliative Care, Inselspital, Bern University Hospital, University of Bern, Freiburgstrasse 28, 3010 Bern, Switzerland; 6 Department of Geriatrics, Inselspital, Bern University Hospital, and University of Bern, Freiburgstrasse, 3010 Bern, Switzerland; 7 The Dartmouth Institute of Health Policy & Clinical Practice, Lebanon, NH USA; 8 Section of Geriatrics, Boston University Medical Center, Boston, MA USA

**Keywords:** End-of-life, Delivery of health care, Health care cost, Health insurance, Switzerland

## Abstract

**Background:**

Lack of health insurance claims (HIC) in the last year of life might indicate suboptimal end-of-life care, but reasons for no HIC are not fully understood because information on causes of death is often missing. We investigated association of no HIC with characteristics of individuals and their place of residence.

**Methods:**

We analysed HIC of persons who died between 2008 and 2010, which were obtained from six providers of mandatory Swiss health insurance. We probabilistically linked these persons to death certificates to get cause of death information and analysed data using sex-stratified, multivariable logistic regression. Supplementary analyses looked at selected subgroups of persons according to the primary cause of death.

**Results:**

The study population included 113,277 persons (46% males). Among these persons, 1199 (proportion 0.022, 95% CI: 0.021–0.024) males and 803 (0.013, 95% CI: 0.012–0.014) females had no HIC during the last year of life. We found sociodemographic and health differentials in the lack of HIC at the last year of life among these 2002 persons. The likelihood of having no HIC decreased steeply with older age. Those who died of cancer were more likely to have HIC (adjusted odds ratio for males 0.17, 95% CI: 0.13–0.22; females 0.19, 95% CI: 0.12–0.28) whereas those dying of mental and behavioural disorders (AOR males 1.83, 95% CI:1.42–2.37; females 1.65, 95% CI: 1.27–2.14), and males dying of suicide (AOR 2.15, 95% CI: 1.72–2.69) and accidents (AOR 2.41, 95% CI: 1.96–2.97) were more likely to have none. Single, widowed, and divorced persons also were more likely to have no HIC (AORs in range of 1.29–1.80). There was little or no association between the lack of HIC and characteristics of region of residence. Patterns of no HIC differed across main causes of death. Associations with age and civil status differed in particular for persons who died of cancer, suicide, accidents and assaults, and mental and behavioural disorders.

**Conclusions:**

Particular groups might be more likely to not seek care or not report health insurance costs to insurers. Researchers should be aware of this aspect of health insurance data and account for persons who lack HIC.

**Electronic supplementary material:**

The online version of this article (10.1186/s12913-018-2984-2) contains supplementary material, which is available to authorized users.

## Background

Health insurance claims (HIC) offer cost-effective research potential for studying large populations to track determinants and variations in the use of healthcare [1, 2]. HIC are particularly important in Switzerland, where population-wide data about healthcare use, particularly relating to cost and end-of-life (EOL) care, are scarce or fragmented [3]. Swiss HIC data inform aspects of healthcare delivery such as EOL cost trajectories [4], the burden of schizophrenia [5], and potentially inappropriate medications [6], just to name a few. Yet despite its strengths, HIC data lack information that otherwise could improve their usefulness in health services research. Information that is important in, for example, EOL studies, on cause of death, is often not readily available [7].

Swiss residents enjoy one of the best performing healthcare systems and have one of the highest life expectancies in the world. At the same time, this system is characterized by high costs and complexity that make it difficult to manage and change. With large choice and wide supply of services, individuals might find it hard to find optimal solutions [8]. Similarly, high spending might not necessarily mean high quality; in 2010 the Economist Intelligence Unit ranked Switzerland 30th out of 40 in quality of end-of-life care [9].

Healthcare expenditures tend to rise, often sharply, near the end of life [4, 10]. Studies often use development of cost over a certain period or aggregate overall cost within a certain period prior to death [11]. With advances in healthcare in general, and the growing intensity of EOL care in particular, these costs tend to be substantial. However, some small proportion of persons die with either no healthcare use at all, or at least no HIC. Lack of HIC in the last year of life might indicate suboptimal EOL care, but the reasons for no HIC are not fully understood. We therefore investigated associations between the lack of end-of-life health insurance claims and characteristics of individuals and where they live.

## Methods

### Study design & data sources

This study used routinely collected Swiss HIC data in a retrospective, secondary analysis of data that are described in detail elsewhere [12]. To summarize, data from six of the 10 largest insurers operating in the Swiss market were pooled and used to track healthcare use over the last year of life of adults who died between 2008 and 2010 [13]. The insurance providers delivered data on sex, date of birth, date of death, place of residence (community or postcode), and complete records of HIC of policyholders. Based on the communities in which they resided, we deterministically linked data on level of urbanization, language region, and neighbourhood socioeconomic status.

Using dates of birth and death, sex, and place of residence, we probabilistically linked the insured persons file to the death certificates in the Swiss Federal Statistical Office’s database (see [12] for details on linkage procedure and results). In addition to causes of death, this data linkage also provided information about civil status and nationality.

### Study setting

Basic health insurance, which covers all services related to illness and pregnancy deemed medically necessary and cost-appropriate, is mandatory for and offered to all Swiss residents with no prior checks or restrictions [14]. Eighty-one private insurers (at the time closest to the end of the study period, 10 August 2011) offered the mandatory basic insurance package. Insurers vary greatly in size and coverage from the 858,005 insured by CSS (which provided data for this study) to 170 persons insured by Krankenkasse Zeneggen [13]. The mean number of those insured by all providers is 96,045 (standard deviation 167,746).

Residents choose a deductible in a range of 300–2500 Swiss Francs (1 CHF = 0.85 Euro = 1.01 US$, as of 25 December 2017); higher deductibles and managed care plans lower the cost of premiums. Social assistance subsidizes premiums for low-income persons. Hospital claims are delivered directly to insurers, whereas a majority of other services is paid by individuals and later reimbursed by insurers after deductibles have been met. Individuals can voluntarily supplement the basic insurance package with private insurance to add further provider and treatment choices (e.g., complementary medicine, dental care) and cover additional benefits (e.g., a private room during a hospital stay). A separate mandatory insurance system covers HIC that are accident related [4, 7, 8, 12, 15].

### Conceptual framework

**Fig. 1:**
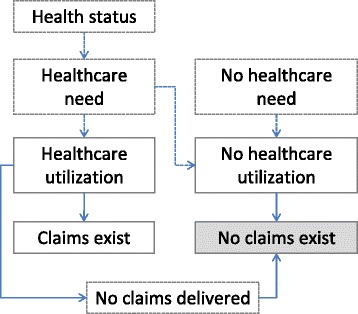
Pathways of healthcare needs and healthcare utilization leading to existence or lack of health insurance claims. Boxes with solid-line boundaries represent observed events; dashed-line boundaries, unobserved, potential events shaping (lack of) utilization

We hypothesized that having no health insurance claims in the last year of life may occur in two main ways: either a person used no healthcare, or healthcare was delivered but no HIC were filed (Fig. 1). In the first case, a person could have died having had no need of healthcare, having had all healthcare needs met by the family, having refused treatment, having been incapable of finding or paying for healthcare, having been undertreated, or a person could have died suddenly with no possibility of medical care. In the second case, care was in fact delivered but for one reason or another information on its cost never reached the insurer. This could happen, for example, in situations in which healthcare was paid entirely out-of-pocket or by supplemental healthcare insurance, the cost of care did not reach the level of the relevant deductible and information about that cost did not reach the insurer, or a patient or caregiver was not willing or capable of handling the HIC documents.

### Analyses

Guided by previous work [12], we stratified analyses by sex. In the last year of life, the absence of reimbursed HIC as opposed to having some HIC was a binary outcome of the analyses. We calculated frequencies of persons with and without HIC and proportions of persons without HIC across covariates. We used logistic regression with robust standard errors that adjusted for clustering of decedents within regions of residence [16]. Multivariable models included age in 5-year bands, nationality (Swiss or foreigner, including unknown), and civil status (single, married, widowed, divorced), and cause of death, which was coded according to the 10th revision of the International Statistical Classification of Diseases and Related Health Problems (ICD-10): cardiovascular diseases (CVD, all I codes), cancer (all C codes), mental and behavioural disorders (all F codes), diseases of the nervous system (all G codes), respiratory diseases (all J codes), diseases of the digestive system (all K codes) accidents and assaults (all V, all W and X00-X59, X85-X99, Y00-Y09, Y85-Y86 codes), suicide (X60-X84 codes), and other (remaining codes). Characteristics of regions of residence included level of urbanization (urban, periurban, rural), language region (German, French, Italian) and quintiles of median area-based socioeconomic position (Swiss-SEP) index [17]. We conducted supplementary analyses by main causes of death to explore whether the associations differed across these strata. These analyses further aggregated age categories in order to have a sufficient number of events across groups.

## Results

The probabilistic linkage to the death certificate database had a 95.6% success rate; unlinked individuals were similar to the linked ones and were excluded. The study population consisted of 113,277 persons (Table 1) who comprised 61.3% of those who died in Switzerland between 2008 and 2010. The sex, age, nationality, civil status, level of urbanization, language region, Swiss-SEP and, most importantly, cause of death distributions were almost identical to overall mortality in that time period [12].

**Table 1:** Study population. Distribution of persons with and without health insurance claims and proportion (and 95% confidence interval) across analysed variables. Attribution of causes of death follows ICD-10 coding

Category	Males					Females				
	HIC exist		No HIC		*Proportion no* HIC *(95% CI)*	HIC exist		No HIC		*Proportion no* HIC *(95% CI)*
	*No.*	*Col %*	*No.*	*Col %*		*No.*	*Col %*	*No.*	*Col %*	
Age group										
*19–25*	253	0%	61	5%	0.194 (0.151–0.238)	105	0%	8	1%	0.071 (0.024–0.118)
*26–30*	214	0%	40	3%	0.157 (0.113–0.202)	126	0%	3	0%	0.023 (0.000–0.049)
*31–35*	262	1%	38	3%	0.127 (0.089–0.164)	153	0%	9	1%	0.056 (0.020–0.091)
*36–40*	384	1%	48	4%	0.111 (0.081–0.141)	252	0%	4	0%	0.016 (0.000–0.031)
*41–45*	671	1%	80	7%	0.107 (0.084–0.129)	391	1%	10	1%	0.025 (0.010–0.040)
*46–50*	1111	2%	93	8%	0.077 (0.062–0.092)	702	1%	14	2%	0.020 (0.009–0.030)
*51–55*	1606	3%	107	9%	0.062 (0.051–0.074)	1048	2%	27	3%	0.025 (0.016–0.034)
*56–60*	2411	5%	123	10%	0.049 (0.040–0.057)	1468	2%	23	3%	0.015 (0.009–0.022)
*61–65*	3672	7%	105	9%	0.028 (0.023–0.033)	2191	4%	32	4%	0.014 (0.009–0.019)
*66–70*	4584	9%	107	9%	0.023 (0.019–0.027)	2801	5%	41	5%	0.014 (0.010–0.019)
*71–75*	5713	11%	85	7%	0.015 (0.012–0.018)	4153	7%	52	6%	0.012 (0.009–0.016)
*76–80*	7925	15%	77	6%	0.010 (0.007–0.012)	6607	11%	84	10%	0.013 (0.010–0.015)
*81–85*	9496	18%	89	7%	0.009 (0.007–0.011)	11,268	19%	125	16%	0.011 (0.009–0.013)
*86–90*	8404	16%	92	8%	0.011 (0.009–0.013)	13,527	23%	159	20%	0.012 (0.010–0.013)
*91+*	5411	10%	54	5%	0.010 (0.007–0.013)	14,366	24%	212	26%	0.015 (0.013–0.016)
Nationality										
*Swiss*	46,768	90%	1013	84%	0.021 (0.020–0.022)	55,671	94%	748	93%	0.013 (0.012–0.014)
*Foreigner*	5349	10%	186	16%	0.034 (0.029–0.038)	3487	6%	55	7%	0.016 (0.011–0.020)
Civil status										
*Single*	6247	12%	380	32%	0.057 (0.052–0.063)	6978	12%	134	17%	0.019 (0.016–0.022)
*Married*	30,452	58%	476	40%	0.015 (0.014–0.017)	13,064	22%	131	16%	0.010 (0.008–0.012)
*Widowed*	10,796	21%	138	12%	0.013 (0.011–0.015)	33,875	57%	425	53%	0.012 (0.011–0.014)
*Divorced*	4622	9%	205	17%	0.042 (0.037–0.048)	5241	9%	113	14%	0.021 (0.017–0.025)
Cause of death										
*CVD*	17,398	33%	353	29%	0.020 (0.018–0.022)	22,858	39%	321	40%	0.014 (0.012–0.015)
*Cancer*	16,107	31%	76	6%	0.005 (0.004–0.006)	13,250	22%	48	6%	0.004 (0.003–0.005)
*Mental & behavioural disorders*	2313	4%	87	7%	0.036 (0.029–0.044)	4670	8%	110	14%	0.023 (0.019–0.027)
*Nervous system*	2178	4%	40	3%	0.018 (0.012–0.024)	3256	6%	66	8%	0.020 (0.015–0.025)
*Respiratory*	3637	7%	39	3%	0.011 (0.007–0.014)	3360	6%	41	5%	0.012 (0.008–0.016)
*Digestive*	2088	4%	35	3%	0.016 (0.011–0.022)	2419	4%	34	4%	0.014 (0.009–0.018)
*Accidents*	1980	4%	208	17%	0.095 (0.083–0.107)	1955	3%	33	4%	0.017 (0.011–0.022)
*Suicide*	1213	2%	162	14%	0.118 (0.101–0.135)	581	1%	15	2%	0.025 (0.013–0.038)
*Other*	5203	10%	199	17%	0.037 (0.032–0.042)	6809	12%	135	17%	0.019 (0.016–0.023)
Urbanicity										
*Urban*	16,086	31%	392	33%	0.024 (0.021–0.026)	20,503	35%	349	43%	0.017 (0.015–0.018)
*Peri-urban*	22,725	44%	528	44%	0.023 (0.021–0.025)	24,819	42%	324	40%	0.013 (0.011–0.014)
*Rural*	13,306	26%	279	23%	0.021 (0.018–0.023)	13,836	23%	130	16%	0.009 (0.008–0.011)
Language region										
*German*	36,616	70%	877	73%	0.023 (0.022–0.025)	42,034	71%	548	68%	0.013 (0.012–0.014)
*French*	12,857	25%	268	22%	0.020 (0.018–0.023)	13,905	24%	237	30%	0.017 (0.015–0.019)
*Italian*	2644	5%	54	5%	0.020 (0.015–0.025)	3219	5%	18	2%	0.006 (0.003–0.008)
Swiss-SEP quintile										
*1st (lowest)*	3807	7%	81	7%	0.021 (0.016–0.025)	3847	7%	40	5%	0.010 (0.007–0.013)
*2nd*	11,480	22%	233	19%	0.020 (0.017–0.022)	12,323	21%	139	17%	0.011 (0.009–0.013)
*3rd*	14,256	27%	312	26%	0.021 (0.019–0.024)	16,128	27%	194	24%	0.012 (0.010–0.014)
*4th*	17,455	33%	451	38%	0.025 (0.023–0.027)	21,196	36%	334	42%	0.016 (0.014–0.017)
*5th (highest)*	5119	10%	122	10%	0.023 (0.019–0.027)	5664	10%	96	12%	0.017 (0.013–0.020)
Total	52,117	100%	1199	100%	0.022 (0.021–0.024)	59,158	100%	803	100%	0.013 (0.012–0.014)

Abbreviations: Col, column; HIC, health insurance claims; CI, confidence interval; Mental & behav., mental and behavioural disorders; Swiss-SEP, Swiss neighbourhood index of socioeconomic position [13]

**Fig. 2:**
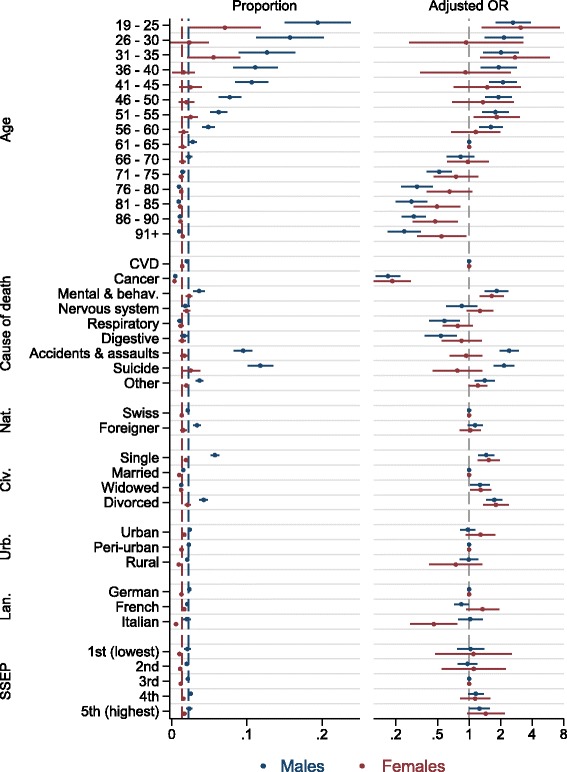
Proportions (left panel) and adjusted odds ratios (AOR, right panel) and their 95% confidence intervals (CI) of lack of health insurance claims. AORs from sex-stratified, multivariable logistic models with robust standard errors. Lack of CI in the left panel indicates very narrow CI. Lack of CI in the right panel indicates reference category (for instance CVD). Dashed lines in the left panel represent sex-specific means. Abbreviations: CVD, cardiovascular diseases; Mental & behave., Mental and behavioural disorders; Nat., nationality; Civ., civil status at the time of death; Urb., level of urbanization; Lan., language region; SSEP, Swiss neighbourhood index of socioeconomic position [13] (in quintiles). Attribution of causes of death follows ICD-10 coding

One thousand one hundred ninety nine males (0.022, 95% confidence interval: 0.021–0.024) and 803 females (0.013, 95% CI: 0.012–0.014) did not have any reimbursed HIC in the last year of life. The proportion of persons with no HIC decreased sharply with age, particularly among males; for example, 0.194 (95% CI: 0.151–0.238) of men who were 19–25 at death had no claim, as opposed to approximately 0.010 (95% CI: 0.007–0.013) of those 76 and older (Fig. 2, left panel). More males dying of accidents and assaults (0.095, 95% CI: 0.083–0.107) and suicide (0.118, 95% CI: 0.101–0.135) had no HIC, whereas the proportion of persons who died of cancer and had no HIC was low (0.005, 95% CI: 0.004–0.006 for men; 0. 004, 95% CI: 0.003–0.005 for women). Slightly more foreign, single, or divorced males had no HIC.

A strong, negative age gradient remained in the multivariable logistic regression model (Fig. 2, right panel; see Additional file 1 for exact estimates of AOR); the adjusted odds ratio (AOR) for the youngest males was 2.63 (95% CI: 1.79–3.86, compared to persons 61–65 years old), whereas for the oldest it was 0.24 (95% CI: 0.17–0.35). In comparison to persons dying from CVD, cancer patients were unlikely to have no HIC (AOR 0.17, 95% CI: 0.13–0.22 for males; 0.19, 95% CI: 0.12–0.28 for females) with weaker effects for young males dying of respiratory and digestive organs diseases. On the other hand, males dying from accidents or assaults (AOR 2.41 95% CI: 1.96–2.97) or suicide (AOR 2.15 95% CI: 1.72–2.69) had higher probability of not having any HIC. For both sexes, those who died of mental and behavioural disorders as well as single, widowed, and divorced persons were more likely to have no HIC. There was little or no association with place of residence apart from a lowered AOR for females in the Italian speaking part of Switzerland.

We found different patterns of association across selected main causes of death among those having no HIC [see Additional file 2]. Persons who died of CVD resembled the overall findings. Persons who died of accidents and assaults showed an association mainly with age. Associations with age and civil status varied the most. For example, no association with age was observed among either persons who died of cancer or females who died of mental and behavioural disorders and suicide. Neither did civil status play a role for males who died of mental and behavioural disorders and suicide.

## Discussion

### Principal findings

We found demographic, health, and socioeconomic differentials in the lack of health insurance claims, and possibly costs, in the last year of life. Several groups of patients identified by sex, age, civil status, and cause of death had a higher probability of not having HIC. Region of residence had little effect, and associations with age and civil status varied for certain causes of death.

### Strengths

This is the first study to the authors’ knowledge to have looked at the lack of mandatory health insurance (MHI) reimbursed healthcare in the last year of life. We used a large, diverse, and representative database of HIC augmented by probabilistic linkage to a database of causes of death and regional characteristics [12]. MHI covers the entire range of providers, including hospital and ambulatory care, medication, and nursing home medical costs.

### Relation to other studies

Unsurprisingly, patterns identified in this study reflect other findings on overall cost of care [12]. For example, persons who died of cancer had higher costs and lower probability of having had no HIC whereas for younger, widowed, and divorced persons the opposite was true. Men and unmarried persons in the U.S. were also found less likely to receive care in the last year of life [18]. Being male, younger, healthier, and living alone was also found to be associated with higher failure to pay insurance premiums in Switzerland [19], and could partly support our findings of either delayed healthcare or lack of healthcare needs in groups with these attributes. Reich et al. showed that persons of lower socioeconomic status, who receive social assistance, generally have higher intensity and cost of healthcare [20], which parallels our weak association of no HIC among individuals from high Swiss-SEP regions. Similarly as in the case of mortality, the use of ecological instead of individual SEP may be a reason for weaker association [17]. The Reich et al. study also identified higher rates of social assistance among those suffering from psychological disorders and psychoses (identified using pharmaceutical cost groups) [20], which supports the idea that these groups might be more economically vulnerable. Mental illness has been associated with problems in paying medical bills [21] and forgone medical and prescription care [22], and these findings parallel results of this study. Finally, a review of international data (excluding Switzerland) estimated that around 23% of persons had no contact with primary care in the last year of life, with lower figures for women than men and for older than younger persons [23]. Our estimates indicate lower proportions, potentially suggesting higher use of healthcare; however, we included any MHI claim, which might lead to an overestimate.

### Implications

Our results suggest that persons with mental and behavioural disorders, those who are prone to suicide, and persons who are unmarried might be more likely to be unable to identify health needs, fail to seek needed healthcare, or to some degree be less able to handle healthcare-related administrative tasks. Married persons, in contrast, might be more likely to have their HIC submitted after death by a spouse. Future research should therefore explore why healthcare is not utilized by particular groups. Researchers and policy makers also should be aware that analyses based only on persons having HIC might have a differential bias that misses certain groups; studies of healthcare costs might choose to log-transform the outcomes and exclude persons with zero cost [24]. Although the lack of cost seems to be rare phenomenon in EOL studies, other aspects of healthcare use might be more affected.

### Limitations

As is also true of other HIC-based studies, this analysis had limited access to individual-level characteristics that could potentially explain observed patterns. For instance, morbidity, functional status, patient preferences, chosen deductibles, and individual socioeconomic position are not available in HIC data yet could influence lack of HIC and should be explored. An estimated 2–3% of all claim invoices are paid by patients directly and never reach their insurers [7]. This could potentially have had an impact on our results, particularly among persons with high deductibles or those dying from causes of death not associated with frequent or expensive healthcare use. Switzerland has a relatively high share of out-of-pocket payments in the last year of life [10], and persons with high deductibles are known to incur lower costs [25]. Though the last year of life is used frequently [9], it is still an arbitrary time frame [26]. Our previous analyses indicate that it performs similarly to the last 3 months of life when analysing costs [12].

## Conclusions

Particular groups might be more likely to not seek care or not report health insurance costs to insurers. Researchers should be aware of this aspect of health insurance data and account for persons who lack HIC.

## Additional files

Additional file 1: Adjusted odds ratios (AOR, right panel) and their 95% confidence intervals (CI) of lack of health insurance claims (as presented on Fig. 2). AORs from sex-stratified, multivariable logistic models with robust standard errors. Abbreviations: CVD, cardiovascular diseases; Mental & behave., Mental and behavioural disorders; Nat., nationality; Civ., civil status at the time of death; Urb., level of urbanization; Lan., language region; Swiss-SEP, Swiss neighbourhood index of socioeconomic position [13]. Attribution of causes of death follows ICD-10 coding. (XLS 33 kb)

Additional file 2: Adjusted odds ratios (AOR) and their 95% confidence intervals (CI) of lack of health insurance claims across selected main causes of death. AORs from sex-stratified, multivariable logistic models with robust standard errors. Lack of CI indicates reference category (for instance CVD). There were no events (having no HIC) among females aged 19–50 dying of mental and behavioural disorders. Abbreviations: CVD, cardiovascular diseases; Mental & behav., Mental and behavioural disorders; Nat., nationality; Civ., civil status at the time of death; Urb., level of urbanization; Lan., language region; Swiss-SEP, Swiss neighbourhood index of socioeconomic position [13]. Attribution of causes of death follows ICD-10 coding. (PDF 82 kb)

## Abbreviations

AOR: Adjusted odds ratio
CI: Confidence interval
CVD: Cardiovascular diseases
EOL: End of life
HIC: Health insurance claims
ICD-10: 10th revision of the International statistical classification of diseases and related health problems
MHI: Mandatory health insurance
Swiss-SEP: Swiss neighbourhood index of socioeconomic position
