# Supplementary figures and images for: Death at no cost? Persons with no health insurance claims in the last year of life in Switzerland
Source: BMC Health Serv Res. 2018 Mar 14;18:178. doi: 10.1186/s12913-018-2984-2 (PMC5853076; doi:10.1186/s12913-018-2984-2)

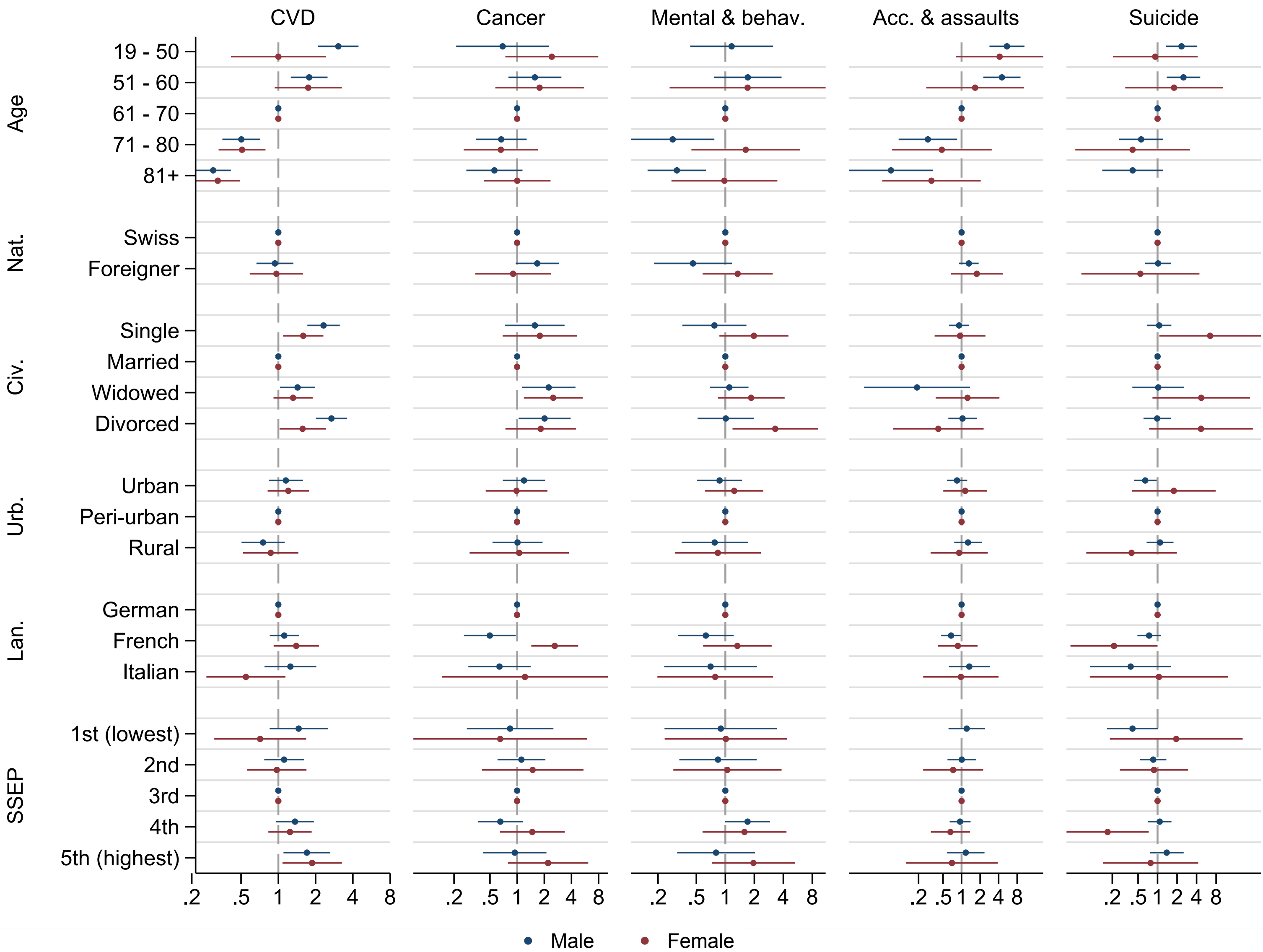

Supplement: Supplementary file 2 — Adjusted odds ratios (AOR) and their 95% confidence intervals (CI) of lack of health insurance claims across selected main causes of death. AORs from sex-stratified, multivariable logistic models with robust standard errors. Lack of CI indicates reference category (for instance CVD). There were no events (having no HIC) among females aged 19–50 dying of mental and behavioural disorders. Abbreviations: CVD, cardiovascular diseases; Mental & behav., Mental and behavioural disorders; Nat., nationality; Civ., civil status at the time of death; Urb., level of urbanization; Lan., language region; Swiss-SEP, Swiss neighbourhood index of socioeconomic position [13]. Attribution of causes of death follows ICD-10 coding. (PDF 82 kb) [file 12913_2018_2984_MOESM2_ESM.pdf]
